# Supplementary figures and images for: Small Nucleolar Derived RNAs as Regulators of Human Cancer
Source: Biomedicines. 2022 Jul 28;10(8):1819. doi: 10.3390/biomedicines10081819 (PMC9404758; doi:10.3390/biomedicines10081819)

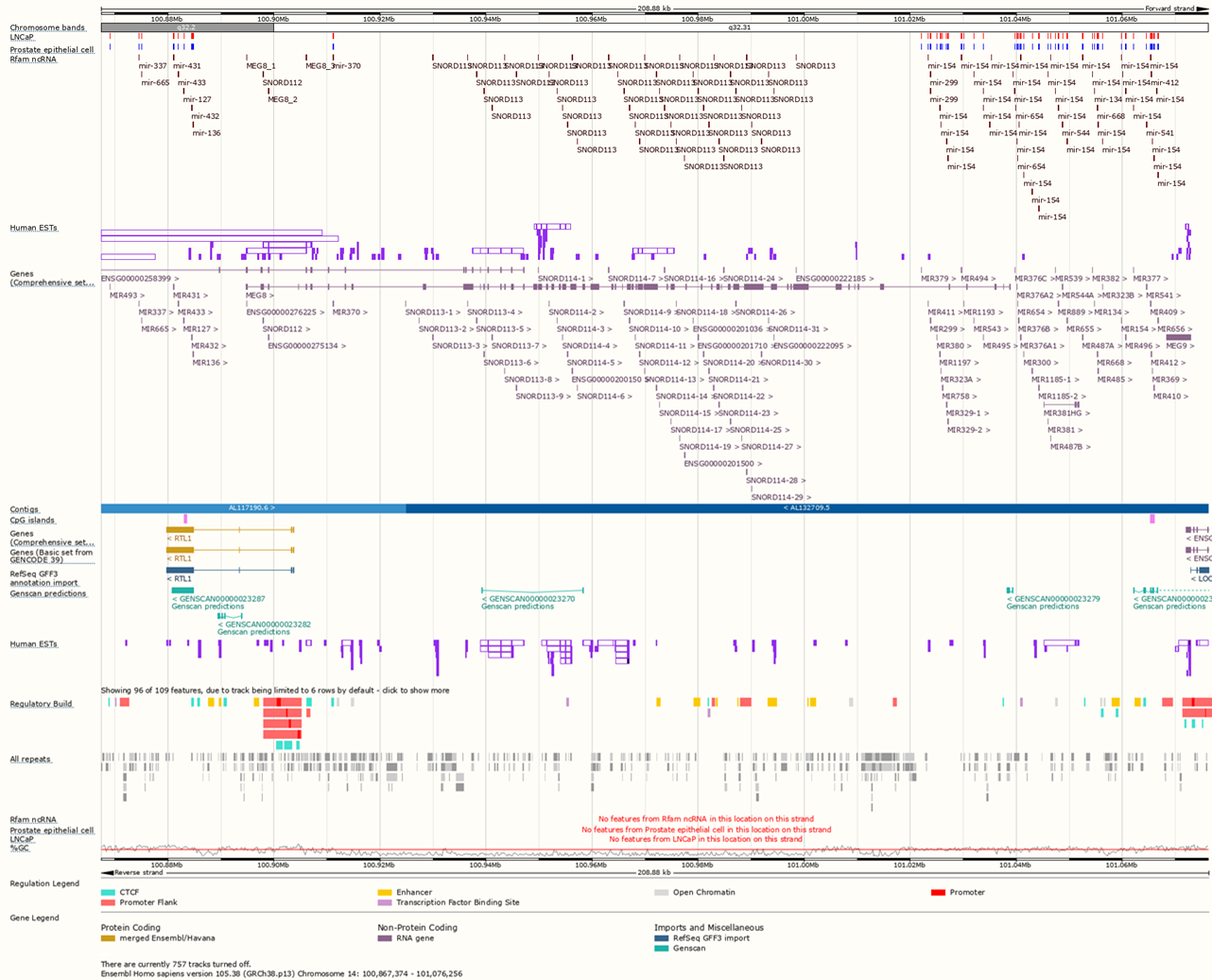

Supplement: Supplementary file 1 [file biomedicines-10-01819-s001.zip › biomedicines-1772706-supplementary.tif]
